# Absorption, metabolism, and excretion of [ ^14^C]dersimelagon, an investigational oral selective melanocortin 1 receptor agonist, in preclinical species and healthy volunteers

**DOI:** 10.1002/prp2.1084

**Published:** 2023-04-20

**Authors:** Minoru Tsuda, Kei Ogawa, Tadashi Endou, Takahiro Goto, Yuko Ogasawara, Akihito Ogasawara

**Affiliations:** ^1^ DMPK Research Laboratories Mitsubishi Tanabe Pharma Corporation Yokohama Japan; ^2^ Data Science Department Mitsubishi Tanabe Pharma Corporation Tokyo Japan

**Keywords:** absorption, dersimelagon, erythropoietic protoporphyria, excretion, metabolism, X‐linked protoporphyria

## Abstract

Dersimelagon (formerly MT‐7117) is a novel, orally administered nonpeptide small molecule selective agonist for melanocortin 1 receptor currently being investigated for the treatment of erythropoietic protoporphyria, X‐linked protoporphyria, and diffuse cutaneous systemic sclerosis (dcSSc). Findings of studies evaluating the absorption, distribution, metabolism, and excretion (ADME) of dersimelagon following a single dose of [^14^C]dersimelagon in healthy adult volunteers (*N* = 6) who participated in phase 1, single‐center, open‐label, mass balance study (NCT03503266), and in preclinical animal models are presented. Rapid absorption and elimination were observed following oral administration of [^14^C]dersimelagon in clinical and nonclinical studies, with a mean *T*_max_ of 30 min in rats and 1.5 h in monkeys, and a median *T*_max_ of 2 h in humans. In rats, there was a widespread distribution of [^14^C]dersimelagon‐related material, but little or no radioactivity was detected in the brain or fetal tissues. In humans, elimination of radioactivity in urine was negligible (excretion of radioactivity into the urine: 0.31% of dose), and the primary route of excretion was feces, with more than 90% of the radioactivity recovered through 5 days postdose. Based on these findings, dersimelagon is not retained in the human body. Findings from humans and animals suggest dersimelagon is extensively metabolized to the glucuronide in the liver, which is eliminated in bile, and hydrolyzed to unchanged dersimelagon in the gut. The results to date for this orally administered agent elucidate the ADME of dersimelagon in human and animal species and support its continued development for the treatment of photosensitive porphyrias and dcSSc.

## Graphical abstract

Dersimelagon (formerly MT‐7117) is a novel, orally administered nonpeptide small molecule selective agonist for melanocortin 1 receptor currently being investigated for the treatment of erythropoietic protoporphyria, X‐linked protoporphyria, and diffuse cutaneous systemic sclerosis. Studies evaluating absorption, distribution, metabolism, and excretion (ADME) demonstrated dersimelagon is extensively metabolized to the glucuronide in the liver, which is eliminated in bile, and hydrolyzed to unchanged dersimelagon in the gut. The primary route of excretion was feces. Major metabolites detected in human feces and plasma as well as postulated metabolic pathways are shown in the accompanying graphical abstract.

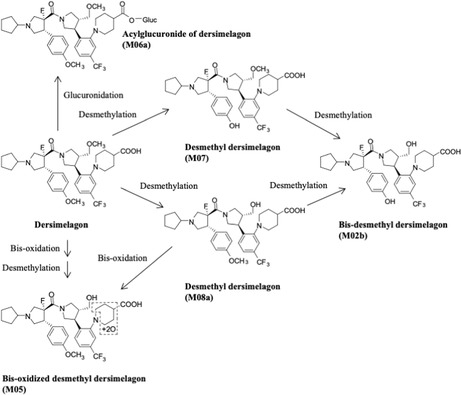

**Abbreviations**
AUC: area under the concentration‐time curve
AUQ: above the upper limit of quantification
BAS: bio‐imaging analyzer system
BLQ: below the lower limit of quantification
BMI: body mass index
*C*_max_
: maximum concentration
dcSSc: diffuse cutaneous systemic sclerosis
EPP: erythropoietic protoporphyria
HPLC: high‐performance liquid chromatography
IV: intravenous
LC‐MS^n^
: liquid chromatography–multistage mass spectrometry
LSC: liquid scintillation counting
MC1R: melanocortin 1 receptor
PK: pharmacokinetic
PPI: proton pump inhibitor
QWBA: quantitative whole‐body autoradiography
SAEs: serious adverse events
TEAEs: treatment‐emergent adverse events
*T*_max_
: time to *C*_max_
XLP: X‐linked protoporphyria

## 1 INTRODUCTION

Erythropoietic protoporphyria (EPP) and X‐linked protoporphyria (XLP) are rare genetic nonacute photosensitive porphyrias resulting from enzyme defects in the heme biosynthetic pathway.^1^, ^2^, ^3^ Symptoms of EPP and XLP first present in early childhood.^2^ The presenting characteristic of EPP and XLP is a burning sensation after sun exposure caused by photoactivation of accumulated protoporphyrin.^1^, ^2^, ^4^ Patients with EPP and XLP can also develop anemia, abnormal serum aminotransferases, cholelithiasis, hepatobiliary disease, and liver failure.^2^, ^5^

Management of EPP and XLP primarily involves avoidance of sunlight with the use of clothing, opaque sunscreens, and other physical measures.^1^, ^2^ Tolerance to sunlight may also be enhanced with oral beta‐carotene.^1^, ^2^ Unfortunately, the need to avoid sunlight impairs quality of life for patients with EPP and XLP.^3^

The melanocortin 1 receptor (MC1R) has emerged as a key pharmacological target for the treatment of patients with EPP and XLP.^4^, ^6^ Multiple lines of evidence indicate that MC1R plays a key role in the production of eumelanin, which is involved in both photoprotection and chemoprotection in response to sunlight exposure.^6^, ^7^

Afamelanotide, an analog of human α‐melanocyte−stimulating hormone, binds to MC1R and increases production of eumelanin.^2^ This agent is approved for treatment of adult patients with EPP and is administered as a subcutaneous implant every 2 months by a trained healthcare professional.^8^ Despite the availability of this injectable treatment for EPP, there remains an unmet need for convenient, efficacious, and safe medicines for this invalidating condition.

### 1.1 Dersimelagon

Dersimelagon (formerly MT‐7117) is a novel, synthetic, orally administered nonpeptide small molecule selective agonist for MC1R being investigated for the treatment of EPP, XLP, and diffuse cutaneous systemic sclerosis. In preclinical studies, dersimelagon exhibited high affinity for human MC1R, with half maximal effective concentration (EC_50_) values in the nanomolar range.^6^ In animal models, dersimelagon stimulated melanin production and increased skin pigmentation.^6^

A phase 1 study (NCT02834442) in healthy adults investigated the safety, tolerability, and pharmacokinetics (PK) of single and multiple ascending doses of dersimelagon. At single doses of 10 mg to 600 mg, median time to maximum concentration (*T*_max_) was ~2 to 5 hours (h) postdose, and mean terminal elimination half‐life (*t*_1/2_) was ~7.6 to 10.6 h. Dersimelagon was rapidly metabolized to dersimelagon glucuronide, a major metabolite of dersimelagon, and demonstrated a PK profile similar to dersimelagon (median *T*_max_ ~ 1 to 5 h); however, the systemic exposure to dersimelagon glucuronide was extremely low compared with that of unchanged dersimelagon (ratio of maximum concentration [*C*_max_] or area under the concentration‐time curve from time 0 extrapolated to infinity [AUC_0‐∞_] of dersimelagon glucuronide to that of unchanged dersimelagon: ≤0.05). Treatment‐related effects on melanin density were observed following multiple doses of 150 mg and 300 mg dersimelagon. In this study, the most commonly reported treatment‐emergent adverse events (TEAEs) were lentigo, skin hyperpigmentation, melanocytic nevus, headache, and ephelides.

Another phase 1 study (NCT03688022) assessed the relative oral bioavailability of two tablet formulations (50 and 100 mg) of dersimelagon and evaluated the effects of gastric conditions (fed, fasted, following administration of a proton pump inhibitor [PPI], with or without consumption of an acidic beverage) on the PK of dersimelagon in healthy adults. Both tablet formulations demonstrated rapid absorption, and the 100‐mg tablets showed a 97% relative oral bioavailability versus 50‐mg tablets, with a slightly lower geometric least squares mean *C*_max_ (12%; 90% CI [0.79–0.98]) observed with the 100‐mg tablets. No clinically relevant effects of administering dersimelagon in fed or fasted conditions or with a PPI were observed.

In phase 2, randomized, placebo‐controlled clinical trial (NCT03520036), dersimelagon at doses of 100 mg and 300 mg increased symptom‐free light exposure and had acceptable tolerability after 16 weeks of treatment in patients with EPP or XLP.^9^

### 1.2 Objectives

To further explore key PK properties of dersimelagon, a series of nonclinical studies and a phase 1 mass balance study (NCT03503266) were conducted. The absorption, distribution, excretion, and metabolism of dersimelagon following a single dose of [^14^C]dersimelagon in healthy adult volunteers and in preclinical animal models were assessed.

## 2 MATERIALS AND METHODS

Nonclinical and clinical studies of [^14^C]dersimelagon are summarized in Table 1. Additional details of the methods are provided in Data S1.

**TABLE 1:** Summary of studies of absorption, distribution, excretion, and metabolism of [^14^C]dersimelagon.

Study type	Species	Sex	Route (dose or concentration)^a^
Nonclinical studies of absorption			
Plasma	Sprague–Dawley albino rat^b^	Male	IV (2 mg/kg), PO (3 mg/kg)
Plasma	Cynomolgus monkey^c^	Male	IV (1 mg/kg), PO (3 mg/kg)
Nonclinical studies of distribution			
QWBA	Sprague–Dawley albino rat^b^	Male	PO (3 mg/kg)
Tissue removal	Long‐Evans pigmented rat^d^	Male	PO (3 mg/kg)
Placental transfer	Pregnant Sprague–Dawley albino rat^b^	Female	PO (3 mg/kg)
Nonclinical studies of excretion			
Urine, feces	Sprague–Dawley albino rat^b^	Male	IV (2 mg/kg), PO (3 mg/kg)
Urine, feces	Cynomolgus monkey^c^	Male	IV (1 mg/kg), PO (3 mg/kg)
Bile, urine, feces	Sprague–Dawley bile duct–cannulated albino rat^b^	Male	PO (3 mg/kg)
Enterohepatic circulation	Sprague–Dawley bile duct–cannulated albino rat^b^	Male	ID (0.121 MBq/body)
Milk secretion	Lactating Sprague–Dawley albino rat^b^	Female	PO (3 mg/kg)
Absorption and excretion in healthy adults			
Plasma, whole blood, urine, feces	Healthy adults	Male	PO (100 mg)
Nonclinical studies of metabolism			
Plasma, urine, feces, bile	Sprague–Dawley albino rat^b^	Male	PO (3 mg/kg)
Plasma, urine, feces	Cynomolgus monkey^c^	Male	PO (3 mg/kg)
Hepatocyte^e^	ICR/CD‐1 mouse^f^	Male	In vitro
Hepatocyte^e^	Sprague–Dawley albino rat^f^	Male	In vitro
Hepatocyte^e^	Cynomolgus monkey^f^	Male	In vitro
Hepatocyte^e^	Human^f^	Mixed	In vitro
Metabolite profiling in healthy adults			
Plasma, feces^g^	Healthy adults	Male	PO (100 mg)

Abbreviations: ID, intraduodenally; IV, intravenously; MBq, megabecquerel; PO, orally; QWBA, quantitative whole‐body autoradiography.

^a^ In all in vivo studies, a single dose was administered.

^b^ Supplied by Charles River Laboratories Japan, Inc., Yokohama, Japan.

^c^ Supplied by Hamri Co., Ltd., Koga City, Japan.

^d^ Supplied by the Institute for Animal Reproduction, Kasumigaura, Japan.

^e^ [^14^C]dersimelagon free base.

^f^ Supplied by BioIVT, Westbury, NY, USA.

^g^ Metabolites in human urine were not analyzed because the cumulative total radioactivity excreted in urine was extremely low.

### 2.1 Test compound ([ ^14^C]dersimelagon)

**FIGURE 1:**
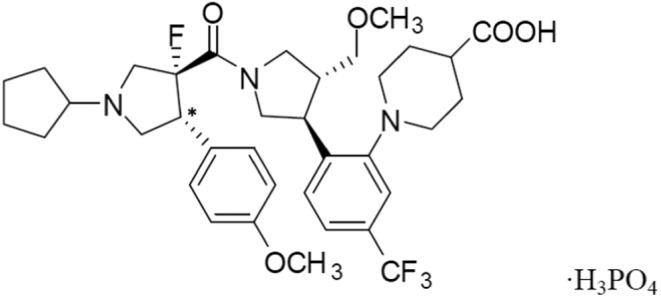
Chemical structure of dersimelagon and labeling position. *^14^C‐labeled position.

Dersimelagon was labeled with ^14^C (Figure 1). Radioactive [^14^C]dersimelagon was prepared by Arcinova (Alnwick, UK). The radiochemical purity and the chemical purity (by ultraviolet detection) of [^14^C]dersimelagon used in the clinical study were 98.7% and 98.3%, respectively. The radiochemical purity of [^14^C]dersimelagon was ≥96% in all nonclinical studies.

### 2.2 Nonclinical studies of absorption, distribution, and excretion of [ ^14^C]dersimelagon

#### 2.2.1 Absorption, distribution, and excretion of [ ^14^C]dersimelagon in rats and monkeys

Absorption was assessed in albino rats and cynomolgus monkeys via radioactivity concentration in plasma following a single oral or intravenous (IV) administration of [^14^C]dersimelagon. Tissue distribution of radioactivity was studied in albino rats by quantitative whole‐body autoradiography (QWBA) following a single oral administration of [^14^C]dersimelagon. In pigmented rats, tissue distribution was studied by tissue removal and subsequent liquid scintillation counting after a single oral administration of [^14^C]dersimelagon. Excretion was measured in urine and feces following a single IV administration and a single oral administration of [^14^C]dersimelagon in intact albino rats and cynomolgus monkeys, and measured in the bile of bile duct–cannulated rats following oral administration.

#### 2.2.2 Placental transfer and milk secretion in female rats

The placental transfer of dersimelagon‐related material in pregnant rats was studied on day 18 of pregnancy following a single oral administration of [^14^C]dersimelagon. The milk secretion of dersimelagon was studied in lactating rats on postpartum day 12.

### 2.3 Phase 1 study in healthy adults

This was a phase 1, single‐center, open‐label, mass balance study (NCT03503266).

#### 2.3.1 Ethics and regulatory compliance

Prior to study initiation, the protocol and all other appropriate documents were reviewed and approved by an independent ethics committee and local regulatory authorities. The study was conducted in accordance with the Declaration of Helsinki, International Conference on Harmonization Good Clinical Practice guidance, applicable regional and local legislation, and standard operating procedures at Clinical Research Services Mannheim GmbH and Mitsubishi Tanabe Pharma Europe Ltd. Written informed consent was obtained from participants prior to the performance of any study‐related assessments and procedures.

#### 2.3.2 Study design

Included participants were White men aged 30–65 years with a body weight of 60–110 kg and body mass index (BMI) of 18–32 kg/m^2^. Participants were healthy and free from illness or disease as determined by medical history, physical examination, electrocardiogram (ECG), vital signs, and laboratory and other tests. Included participants had normal blood pressure, regular daily bowel movements, and agreed to use contraception throughout the study.

Exclusion criteria included current or recent use of food supplements or over‐the‐counter medicines (other than acetaminophen), clinically significant physical or mental illness, abnormal liver enzyme values or creatinine clearance, abnormal ECG findings, surgical or medical conditions that might significantly alter the pharmacokinetics of drugs, drug abuse, excessive alcohol use, use of tobacco products, and recent significant radiation exposure.

The screening visit took place on day −28 to day −2, confinement in the Labcorp Drug Development (formerly Covance) Phase 1 Unit extended from day −1 to day 8, and a follow‐up visit took place on day 15. Participants remained in the unit up to day 14 if it was anticipated by day 8 that ≥90% mass balance of the administered radioactive dose would not be recovered or <1% of the radioactive dose in two consecutive 24‐hour collections of urine and feces had not been achieved.

On day 1, participants received a single oral dose of 100 mg (as free base) [^14^C]dersimelagon containing target radioactivity of 2.2 MBq. Blood samples for total radioactivity, dersimelagon, and metabolite identification were taken predose, at 1, 2, 3, 4, 6, 8, 10, 12, 24, 36, and 48 h postdose; and then every 24 h up to 264 h postdose or sufficient recovery. Urine and feces were collected at the following time intervals: day −1 (−24 h to 0 h) and during 24‐hour intervals postdose up to 264 h or sufficient recovery.

#### 2.3.3 Study objectives

Primary objectives were to assess the pharmacokinetics of total radioactivity in plasma and whole blood, and to assess the excretion of total radioactivity in urine and feces. Secondary objectives were to assess the safety and tolerability of a single dose of [^14^C]dersimelagon. Exploratory objectives included assessment of the metabolic profiles in plasma, urine, and feces, and characterization of the chemical structure of the metabolites.

#### 2.3.4 Pharmacokinetic assessments

The total radioactivity equivalent concentrations in plasma and whole blood (ng eq. of dersimelagon/mL) were summarized and plotted at each sampling time point. PK parameters were derived for plasma and whole blood by noncompartmental analysis using Phoenix WinNonlin® software version 6.3 (Certara, LP, Princeton, NJ, USA). For the calculation of PK parameters, data below the limit of quantification (BLQ) were imputed as a value of zero. PK parameters evaluated included *C*_max_, *T*_max_, AUC, apparent *t*_1/2_, and terminal elimination rate constant (K_el_). Excretion of total radioactivity in urine and feces was expressed as a percentage of radioactivity in the administered dose.

#### 2.3.5 Safety assessments

Safety assessments included TEAEs, laboratory parameters, vital signs, ECG parameters, and physical examinations.

#### 2.3.6 Statistical analyses

No formal statistical tests were performed.

### 2.4 Nonclinical studies of metabolism of [ ^14^C]dersimelagon

Metabolites in plasma, urine, and feces obtained from albino rats and cynomolgus monkeys following a single oral administration of [^14^C]dersimelagon (as well as metabolites in the rat bile) were analyzed using a high‐performance liquid chromatography (HPLC)–radioactivity detection system and liquid chromatography–multistage mass spectrometry (LC‐MS^n^) system.

Metabolism of [^14^C]dersimelagon free base was analyzed in vitro in mouse, rat, monkey, and human hepatocytes by HPLC–radioactivity detection.

### 2.5 Metabolism of [ ^14^C]dersimelagon in healthy adults

Metabolites of dersimelagon were identified in plasma and feces obtained from participants in the phase 1 study using radio–HPLC chromatograms and analyzed using an LC‐MS^n^ system. Based on structural analysis of the metabolites, the postulated metabolic pathways of dersimelagon were constructed.

### 2.6 Nomenclature of target

The key protein target in this article is hyperlinked to the corresponding entry in http://www.guidetopharmacology.org, the common portal for data from the IUPHAR/BPS Guide to PHARMACOLOGY, and is permanently archived in the Concise Guide to PHARMACOLOGY 2019/20.^10^, ^11^

## 3 RESULTS

### 3.1 Pharmacokinetics of [ ^14^C]dersimelagon in rats and monkeys

Following oral dosing of [^14^C]dersimelagon, radioactivity was rapidly absorbed in both rats (*T*_max_, 0.5 h) and monkeys (*T*_max_, 1.5 h; Table S1). Radioactivity declined rapidly in rats (*t*_1/2_, 3.86 h) and more slowly in monkeys (*t*_1/2_, 21.40 h).

### 3.2 Distribution of [ ^14^C]dersimelagon in rats

#### 3.2.1 Distribution in rats

In male albino rats, radioactivity was absorbed rapidly and distributed throughout the body following oral administration of [^14^C]dersimelagon (Table S2). Maximum radioactivity concentrations were generally reached at 30–120 min after dosing; concentrations in the small and large intestines peaked at 4 and 8 h, respectively. The highest levels of radiolabeled material were detected in the liver, followed by the adrenal gland, kidneys, and mesenteric lymph nodes. Radioactivity concentrations in tissues other than the gastrointestinal tract were 2.52 times or less than that in plasma. Radioactivity concentrations declined to BLQ in all tissues except the digestive tract by 24 h postdose, and no detectable level of radioactivity was observed in the brain at any time.

In rats with pigmented skin, tissue distribution and elimination of [^14^C]dersimelagon in the liver and kidney were similar to that observed in albino rats (Table S2). Radioactivity concentration in pigmented skin peaked at 30 min after dosing and declined at a similar rate to that in white (albino) skin. Radioactivity distributed poorly into the eyeball and declined to 10% of maximum radioactivity concentration at 168 h after administration.

#### 3.2.2 Placental transfer in pregnant rats

In a study of placental transfer, radioactivity concentrations in maternal tissues reached maximum concentrations 30 minutes after dosing and declined at a rate similar to that in plasma (Table S3). Radioactivity was distributed to the maternal procreative tissues such as mammary gland, ovary, placenta, and uterus with a level similar to blood. In fetal tissues, little or no radioactivity was detected.

### 3.3 Excretion of [ ^14^C]dersimelagon in rats and monkeys

#### 3.3.1 Excretion in intact rats and monkeys

Following oral or IV administration of [^14^C]dersimelagon, the main route of excretion in intact rats and monkeys (regardless of dosing route) was feces, which accounted for >95% of excreted radioactivity (Table S4). Excretion of radioactivity was almost complete by 96 h in rats and 168 h in monkeys. For intact rats, no radioactivity was excreted in expired air, nor was it detected during cage washing.

#### 3.3.2 Excretion in bile duct–cannulated rats

Following oral administration of [^14^C]dersimelagon to bile duct–cannulated rats, the main route of excretion was bile (63.6% of dose), followed by feces (33.3% of dose; Table S4), indicating that the main route of elimination was fecal excretion via bile. The fraction absorbed from the digestive tract was ~64% based on cumulative radioactivity excreted in bile, urine, and carcass after oral dosing. Following intraduodenal injection of pooled radioactive bile collected from bile duct–canulated rats, excretion of radioactivity was 39.4% in bile, 0.1% in urine, and 59.6% in feces up to 48 h after injection, indicating that approximately 40% of radioactivity excreted in bile is reabsorbed.

#### 3.3.3 Milk secretion in lactating rats

After oral administration of [^14^C]dersimelagon to lactating rats, *C*_max_ in plasma and milk was reached at 30 min. The ratio of radioactivity concentration in milk to plasma was 0.21 at 30 min following administration; this ratio increased to 0.82 at 8 h. The ratio of AUC from the time of dosing to the last time point with a quantifiable concentration of radioactivity concentration in milk to that of plasma was 0.41 (data shown only in text).

### 3.4 Disposition and characteristics of healthy adults

Six participants were enrolled in the phase 1 study, received a single dose of [^14^C]dersimelagon, and completed the study. One participant remained in the unit until day 8, four participants until day 10, and one participant until day 12. All participants were White males. Mean (SD) age was 52.0 (9.5) years, mean (SD) body weight was 86.97 (14.05) kg, and mean (SD) BMI was 28.50 (2.42) kg/m^2^.

### 3.5 Pharmacokinetics of [ ^14^C]dersimelagon in healthy adults

**FIGURE 2:**
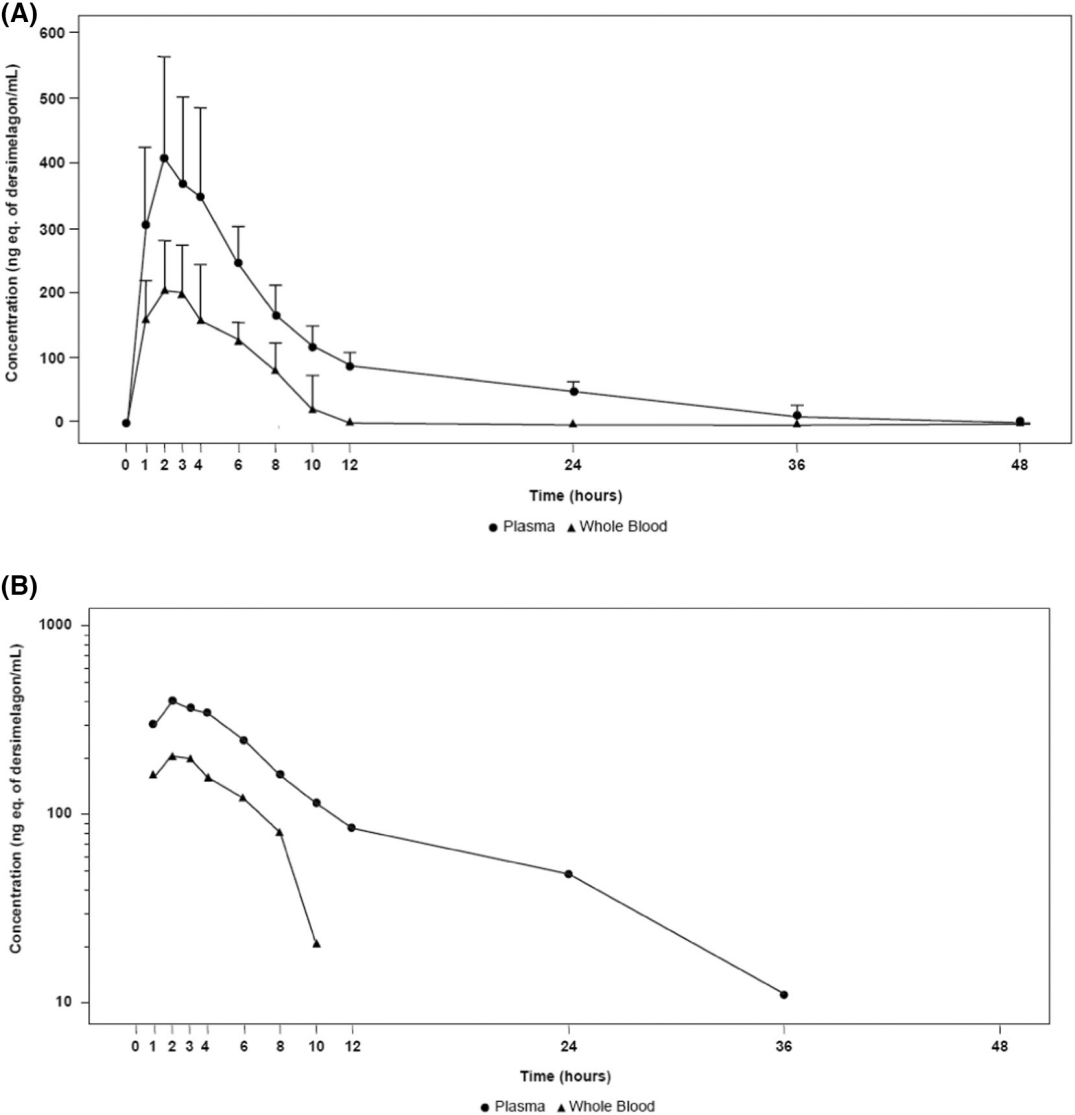
Mean total radioactivity concentration (ng eq. of dersimelagon/mL) in plasma and whole blood in the first 48 h are shown on (A) linear scale (with SD) and (B) semilogarithmic scale following oral administration of [^14^C]dersimelagon in healthy adults.

Among participants in the phase 1 study, both the plasma and whole‐blood concentration‐versus‐time profiles were characterized by a fast absorption phase, with a median *T*_max_ of 2 h following a single oral dose of [^14^C]dersimelagon (Table 2). Following *T*_max_, concentrations showed a rapid decrease with a mean *t*_1/2_ of 12.70 h in plasma and 15.73 h in whole blood. The concentration of total radioactivity in whole blood remained lower than the concentration in plasma at all time points (Figure 2).

**TABLE 2:** Summary of pharmacokinetic parameters for dersimelagon following oral dosing of [^14^C]dersimelagon in healthy adults.

Pharmacokinetic parameter	Plasma total radioactivity	Whole blood total radioactivity
*C*_max_ (ng/mL)^a^	432.2 (151.2)	219.0 (72.2)
*T*_max_ (h)	2.00 (1.00, 4.02)	2.00 (1.07, 4.02)
AUC_0–t_ (ng·h/mL)^a^	3754 (1163)	1158 (440)
AUC_0–∞_ (ng·h/mL)^a^	4462 (1063)	3311 (2268)
*t*_1/2_ (h)	12.70 (5.32)	15.73 (21.43)
*K*_el_ (/h)	0.06 (0.02)	0.11 (0.07)

*Note*: Arithmetic mean (SD) is presented for all variables except *T*_max_, for which median (range) is presented.

Abbreviations: AUC, area under the concentration‐time curve; AUC_0 − ∞_, AUC from time 0 extrapolated to infinity; AUC_0 − t_, AUC from time 0 to the time of the last quantifiable concentration; *C*_max_, maximum observed concentration; h, hours; K_el_, terminal elimination rate constant; *t*_1/2_, apparent terminal elimination half‐life; *T*_max_, time to *C*_max_.

^a^ Units for total radioactivity AUCs and *C*_max_ are ng equivalents·h/mL and ng equivalents/mL, respectively.

### 3.6 Excretion of [ ^14^C]dersimelagon in healthy adults

**FIGURE 3:**
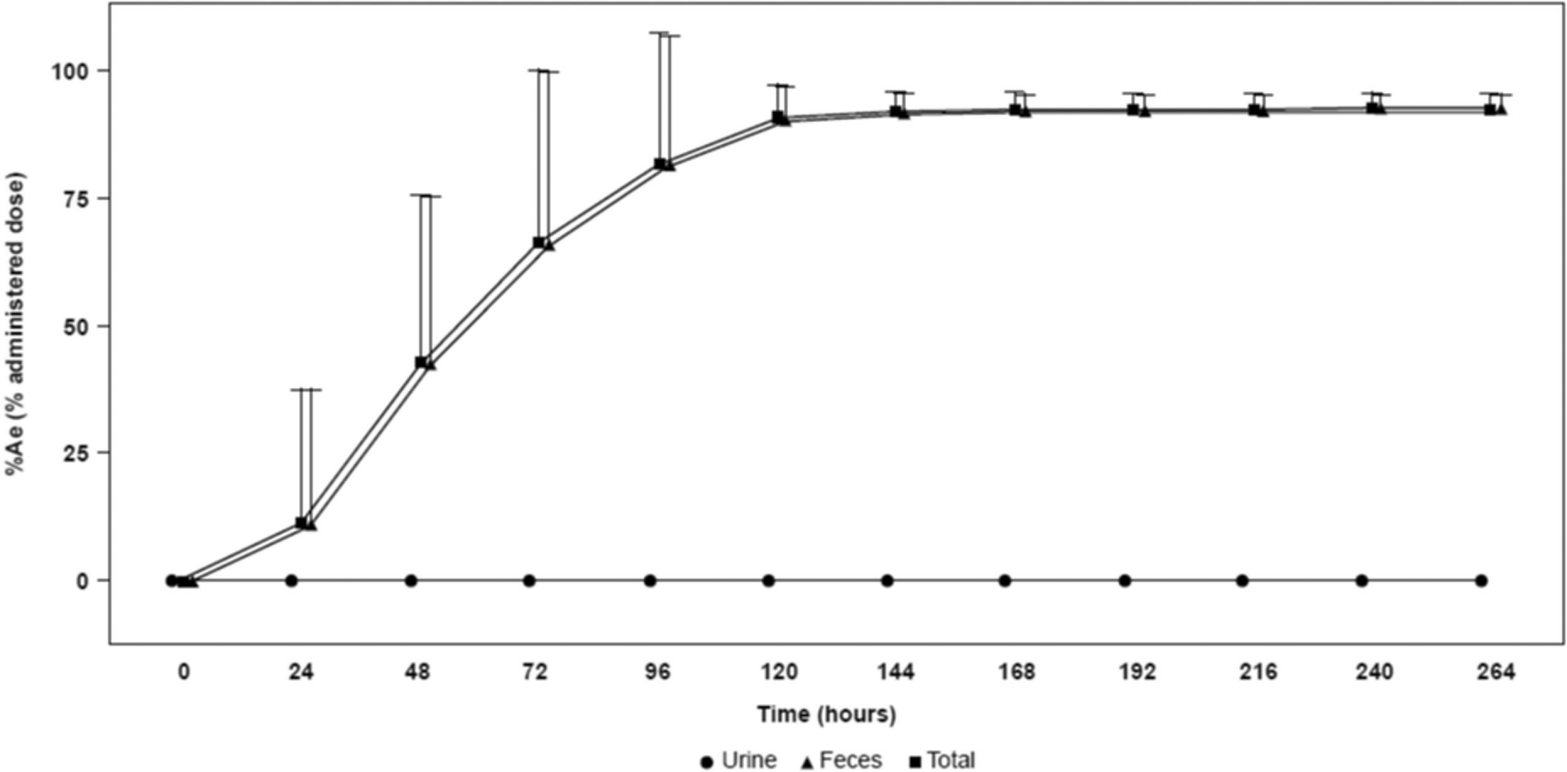
Mean (SD) cumulative excretion of total radioactivity (urine, feces, and total) following oral administration of [^14^C]dersimelagon in healthy adults. %Ae, percentage of amount excreted.

From 0 to 264 h postdose, mean radioactivity recovered in the urine and feces of healthy adults was 0.31% and 92.24% of dose, respectively (total radioactivity recovery, 92.55%; Figure 3). Elimination of radioactivity in urine reached 0.31% at 24 h and was undetectable in urine collections thereafter. Mean recovery of radioactivity in feces reached 65.88% at 72 h, 90.30% at 120 h, and 92.24% at 264 h postdose (Figure 3). From 192 to 216 h postdose, radioactivity was undetectable in feces for four participants and was ≤0.05% for two participants.

### 3.7 Metabolism of [ ^14^C]dersimelagon in rats and monkeys

Metabolites identified in nonclinical and clinical studies are described in Table 3. In vivo studies of the metabolism of [^14^C]dersimelagon found dersimelagon was the major component in the plasma and feces of rats and monkeys (Table S5). The metabolite M06a was a minor component in the plasma of both rats and monkeys.

**TABLE 3:** Metabolites of [^14^C]dersimelagon and [^14^C]dersimelagon free base detected in clinical and nonclinical studies.

Metabolite name	Molecular formula [M + H]^+^	Structure
M01	C_40_^14^CH_52_N_3_O_11_F_4_	Glucuronide of desmethyl dersimelagon
M02a	C_40_^14^CH_52_N_3_O_11_F_4_	Glucuronide of desmethyl dersimelagon
M02b	C_33_^14^CH_42_N_3_O_5_F_4_	Bis‐desmethyl dersimelagon
M02c‐1	C_29_^14^CH_36_N_3_O_5_F_4_	Descyclopentanyl desmethyl dersimelagon
M02c‐2	C_29_^14^CH_36_N_3_O_5_F_4_	Descyclopentanyl desmethyl dersimelagon
M02d	C_41_^14^CH_54_N_3_O_12_F_4_	Glucuronide of oxidized dersimelagon (at cyclopentenyl moiety)
M03a	C_40_^14^CH_52_N_3_O_11_F_4_	Glucuronide of desmethyl dersimelagon
M03b	C_41_^14^CH_54_N_3_O_12_F_4_	Glucuronide of oxidized (at cyclopentanyl moiety) dersimelagon
M04	C_41_^14^CH_54_N_3_O_12_F_4_	Glucuronide of oxidized (at cyclopentanyl moiety) dersimelagon
M05	C_35_H_44_N_3_O_7_F_4_	Bis‐oxidized desmethyl dersimelagon
M06a	C_41_^14^CH_54_N_3_O_11_F_4_	Acyl glucuronide of dersimelagon
M06b	C_35_^14^CH_48_N_3_O_6_F_4_	Hydrated dersimelagon
M06c	C_41_^14^CH_54_N_3_O_11_F_4_	Acyl glucuronide of dersimelagon (acyl migration isomer)
M07	C_34_^14^CH_44_N_3_O_5_F_4_	Desmethyl dersimelagon
M08a	C_34_^14^CH_44_N_3_O_5_F_4_	Desmethyl dersimelagon
M08b	C_35_^14^CH_46_N_3_O_6_F_4_	Oxidized dersimelagon
M09a	C_30_^14^CH_38_N_3_O_5_F_4_	Descyclopentanyl dersimelagon
M09b‐1	C_35_^14^CH_46_N_3_O_6_F_4_	Oxidized dersimelagon
M09b‐2	C_35_^14^CH_46_N_3_O_6_F_4_	Oxidized dersimelagon
M10	C_35_^14^CH_46_N_3_O_6_F_4_	Oxidized dersimelagon
M11	C_35_^14^CH_46_N_3_O_6_F_4_	Oxidized dersimelagon
Dersimelagon	C_35_^14^CH_46_N_3_O_5_F_4_	Dersimelagon
M12	C_35_^14^CH_46_N_3_O_6_F_4_	Oxidized dersimelagon

Similar to the case for plasma, dersimelagon was also a major component of radioactivity in the urine of rats and monkeys. Although other metabolites composed substantial proportions of the radioactivity in urine, these metabolites each accounted for ≤0.1% of dose.

In the feces of rats, dersimelagon was the most abundant component. M07 accounted for 7.4% of radioactivity and 6.8% of dose (Table S5). In monkeys, dersimelagon in feces accounted for just over half (50.4%) of the dose. M08a and M07 accounted for 11.9% and 9.8% of the dose, respectively. In the bile of rats over 0–48 h postdose, the metabolite M06a accounted for 51.0% of the dose, and dersimelagon accounted for 5.2% of the dose (Table S5).

### 3.8 Metabolism of [ ^14^C]dersimelagon free base in hepatocytes

Table S6 summarizes the results of in vitro analyses of the metabolism of [^14^C]dersimelagon‐free base in the hepatocytes of mice, rats, monkeys, and humans. M06a was a major metabolite in all species. Minor metabolites including M02a, M02b, and M08a were also detected (Table S6).

### 3.9 Metabolism of [ ^14^C]dersimelagon in healthy adults

**FIGURE 4:**
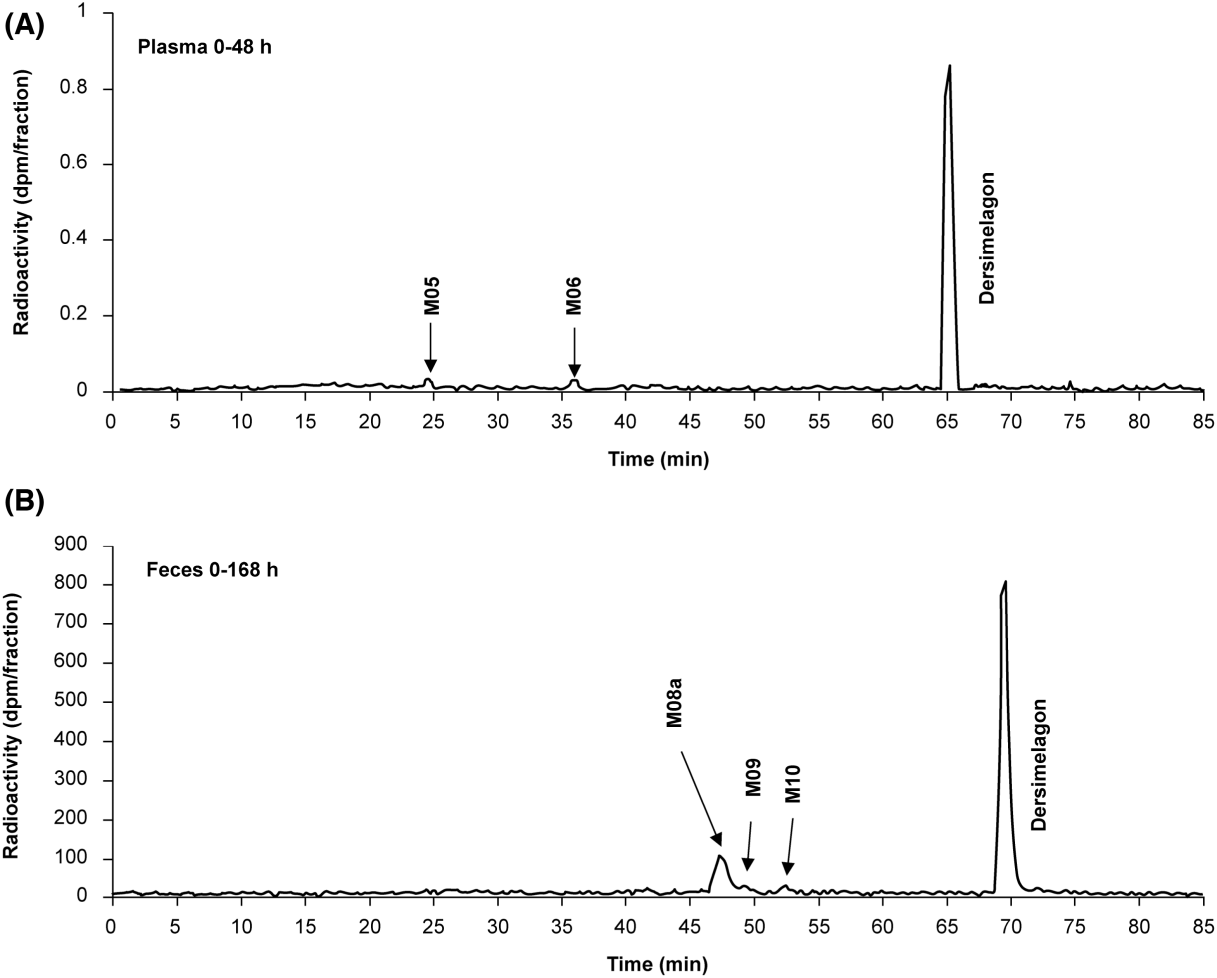
Representative radio–high‐performance liquid chromatography chromatograms of dersimelagon and metabolites in (A) human plasma (0–48 h) and (B) feces (0–168 h) following a single oral dose of [^14^C]dersimelagon in healthy adults. See Tables 3 and 4 for a further description of metabolites. h, hours; min, minutes.

Metabolites in human urine were not analyzed because the radioactivity excreted in urine was extremely low. On radiochromatograms of plasma and feces of human participants following a single oral dose of [^14^C]dersimelagon, dersimelagon was the major component in both plasma and feces, and additional metabolites were identified (Figure 4).

**FIGURE 5:**
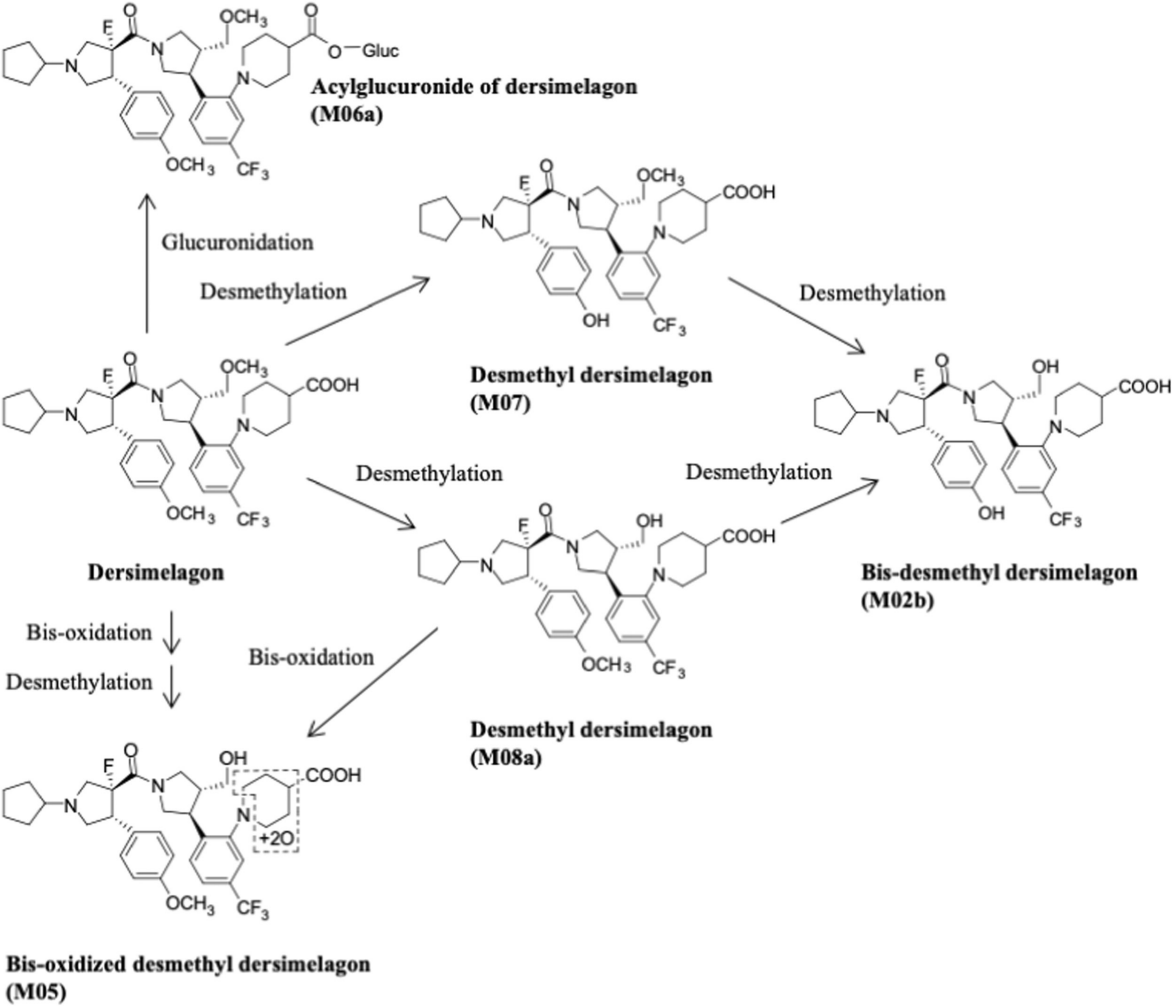
Major metabolites detected in human feces and plasma following oral administration of [^14^C]dersimelagon in healthy adults and postulated metabolic pathways. Metabolites in human urine were not analyzed because the cumulative total radioactivity excreted in human urine was extremely low.

In the plasma of human participants 0–48 h postdose, dersimelagon accounted for 87.3% of radioactivity (Table 4). Metabolites M05 and M06 were detected as minor components in plasma, and no metabolite in plasma accounted for more than 10% of drug‐related exposure. In feces collected 0–168 h postdose, dersimelagon accounted for 64.5% of the dose, and M08a accounted for 10.3% (Table 4). The postulated metabolic pathways of dersimelagon are shown in Figure 5.

**TABLE 4:** Metabolites detected in plasma (0–48 h) and feces (0–168 h) following oral administration of [^14^C]dersimelagon in healthy adults.

Metabolite	Plasma (% total drug‐related exposure)	Feces (% of radioactivity [% of dose])
M05	2.2	–
M06 (M06a + M06b)	2.0	–
M08a	–	11.2 (10.3)
M09 (M09a + M09b)	–	1.3 (1.2)
M10	–	1.4 (1.3)
Dersimelagon	87.3	70.1 (64.5)

*Note*: Metabolites in human urine were not analyzed because the cumulative total radioactivity excreted in human urine was extremely low.

Abbreviation: h, hours.

### 3.10 Safety of [ ^14^C]dersimelagon in healthy adults

Of six participants in the phase 1 study, one (16.7%) participant experienced one TEAE of back pain that was mild in severity and was not considered to be related to study drug. No deaths or serious adverse events (SAEs) were reported. There were no findings of note for any safety laboratory parameters, vital signs, ECG parameters, or physical examination findings.

## 4 DISCUSSION

The absorption, distribution, excretion, and metabolism of dersimelagon were investigated in a series of nonclinical studies and a phase 1 study in healthy adults. All analyses were conducted following a single oral or IV dose of [^14^C]dersimelagon (or [^14^C]dersimelagon free base).

### 4.1 Pharmacokinetics of [ ^14^C]dersimelagon

Radioactivity was rapidly absorbed in humans, rats, and monkeys following oral dosing of [^14^C]dersimelagon, consistent with findings of the earlier phase 1 studies and nonclinical studies in animal species. In humans, the concentration of radioactivity in whole blood was lower than the concentration in plasma (in vitro blood cell distribution was <15%; unpublished data on file, Mitsubishi Tanabe Pharma Corporation, Tokyo, Japan). Radioactivity declined rapidly in all species, with *t*_1/2_ < 24 h in all models. The longer half‐life of dersimelagon in monkeys compared with rats may be related to the contribution of enterohepatic circulation to the elimination of dersimelagon, although a direct comparison between species is not possible because biliary excretion data in monkeys are not available.

Distribution was studied in rats by QWBA and measurement of radioactivity concentrations in removed tissues. In albino rats, the organs with the highest level of radiolabeled material were the liver, followed by the adrenal gland, kidney, and mesenteric lymph nodes. Radioactivity concentrations declined to BLQ in all tissues (except the digestive tract) by 24 h postdose, and radioactivity was not detected in the eyes or brain at any time. Some in vitro studies indicate that dersimelagon is a human P‐glycoprotein substrate, so its brain penetration in humans also may be predicted to be low (unpublished data on file, Mitsubishi Tanabe Pharma Corporation, Tokyo, Japan). In pigmented rat skin, the radioactivity profile was similar to that in nonpigmented skin, suggesting that dersimelagon has little affinity for melanin. Radioactivity appeared higher (though still low) in the eyeballs of pigmented rats compared with albino rats. These varying findings may be attributed to the different methods for measuring tissue radioactivity concentrations in the albino and pigmented rat studies, although it remains possible that a small association of dersimelagon with melanin‐containing tissues was observed. Overall in rats, no tissues or organs had substantial residual radioactivity.

In preclinical studies, the main route of excretion of radioactivity was feces in intact rats and monkeys, and bile in bile duct–cannulated rats. Excretion of radioactivity was almost complete by 4 and 7 days in rats and monkeys, respectively. Little or no radioactivity was detected in fetal tissues following administration to pregnant rats, and [^14^C]dersimelagon‐related material was transferred into the milk of lactating rats. In humans, more than 90% of radioactivity was recovered through 5 days postdose, almost entirely in feces, confirming the main route of excretion. Based on these findings, dersimelagon is excreted almost exclusively via feces, mainly derived from biliary elimination, and is not retained in the human body.

### 4.2 Metabolism of [ ^14^C]dersimelagon

The acylglucuronide of dersimelagon (M06a) was the major metabolite in hepatocyte incubations from humans and animals and was also seen at high levels in rat bile. Given that notably higher levels of M06a than dersimelagon were observed in the rat bile, and unchanged dersimelagon was primarily observed in feces, it is likely that M06a is broken down to dersimelagon in the gut after elimination in bile. Therefore, the extent of metabolism of dersimelagon in rats may be substantially greater than suggested by the metabolite profile in feces. The extent of biliary excretion and the metabolite profiles in bile are not known in monkeys and humans. However, the results of in vitro and in vivo studies of metabolism and excretion in monkeys and humans may suggest that dersimelagon, absorbed from the gut of monkeys and humans, is extensively metabolized to M06a in the liver and eliminated in bile, and then hydrolyzed to unchanged dersimelagon in the gut. Taken together, these findings suggest that the main route of metabolism of dersimelagon in all species is glucuronidation.

In human plasma, dersimelagon was the major component (87% of total exposure), and two metabolites (bis‐oxidized desmethyl dersimelagon [M05] and M06) were observed as minor components (2% of total exposure each). In human feces, dersimelagon was the major component (70% of total exposure) and desmethyl dersimelagon (M08a) was a major fecal metabolite (11% of total exposure); M05 and M06 were not detected. The postulated metabolic pathways of dersimelagon have been constructed.

M08a was also observed as a main metabolite in monkey feces; however, this metabolite was not observed in human or monkey plasma. There is little concern regarding the desmethyl metabolite for safety or drug–drug interactions because there is no exposure in the body, and the amount of excretion in feces is similar between humans and monkeys (constituting only approximately 10% of the dose). Of note, each metabolite observed in human plasma constituted <10% of total drug‐related exposure. Based on the results of all studies presented, the risk of drug–drug interactions with dersimelagon as a victim drug is considered low because dersimelagon is metabolized by multiple metabolic pathways, including glucuronidation and oxidation reactions.

### 4.3 Safety in humans

A single dose of 100 mg [^14^C]dersimelagon was well tolerated by healthy male adults, with only one TEAE reported. No deaths, SAEs, or TEAEs leading to withdrawal occurred during the study. No clinically significant vital signs, ECGs, laboratory values, or physical examination findings were reported.

## 5 CONCLUSIONS

Rapid absorption and elimination were observed following oral administration of [^14^C]dersimelagon in clinical and nonclinical studies. The primary route of excretion was feces, and dersimelagon‐related components were not retained in tissues and organs. Unchanged dersimelagon was a main component in animal and human plasma, and no single metabolite exceeded 10% of drug‐related exposure in human plasma, although dersimelagon was extensively metabolized to the glucuronide in the liver. The results to date for this orally administered agent help to clarify the PK and safety profiles of dersimelagon and support its continued development for the treatment of photosensitive porphyrias.

## AUTHOR CONTRIBUTIONS

Concept and design (MT); acquisition of data (KO, TE, TG, YO); analysis and interpretation of data (MT, KO, AO); all authors contributed to the drafting and critical revision of the manuscript for important intellectual content.

## CONFLICT OF INTEREST STATEMENT

This study was sponsored by Mitsubishi Tanabe Pharma Corporation. All authors are employees of Mitsubishi Tanabe Pharma Corporation.

## Supporting information


Data S1.

## Data Availability

The datasets generated during and/or analyzed in this study are available from the corresponding author upon reasonable request.
