# Supplementary material for: Absorption, metabolism, and excretion of [ 14C]dersimelagon, an investigational oral selective melanocortin 1 receptor agonist, in preclinical species and healthy volunteers
Source: Pharmacol Res Perspect. 2023 Apr 20;11(3):e01084. doi: 10.1002/prp2.1084 (PMC10116397; doi:10.1002/prp2.1084)
Supplement: Supplementary file 1 — Data S1. [file PRP2-11-e01084-s001.docx]

**Supplemental Material for:**

**Absorption, Metabolism, and Excretion of [^14^C]Dersimelagon, an Investigational Oral Selective Melanocortin 1 Receptor Agonist, in Preclinical Species and Healthy Volunteers**

Minoru Tsuda, Kei Ogawa, Tadashi Endou, Takahiro Goto, Yuko Ogasawara, Akihito Ogasawara

**Supplementary Methods**

Animal studies were reviewed and approved by the Institutional Animal Care and Use Committee of Sekisui Medical Co., Ltd., Tokyo, Japan, and were conducted in accordance with the Law for the Humane Treatment and Management of Animals, Standards Relating to the Care and Management of Laboratory Animals and Relief of Pain, Guidelines on Methods of Sacrificing Laboratory Animals, Basic Policies for the Conduct of Animal Experimentation, and Guidelines for Proper Conduct of Animal Experiments.

Nonclinical studies are summarized in **Table 1**.

**Absorption, Distribution, and Excretion in Rats and Monkeys**

*Rats*

- Male Sprague-Dawley (Crl:CD) albino rats aged 6–7 weeks supplied by Charles River Laboratory Japan, Inc., were used to evaluate absorption, distribution, and excretion following a single oral or intravenous (IV) dose of [^14^C]dersimelagon
- A single dose of [^14^C]dersimelagon was administered orally (3 mg/kg) into the stomach via a syringe equipped with oral tube or intravenously (2 mg/kg) via injection into the tail vein
  - Blood was withdrawn serially from the jugular vein of albino rats (n = 8) up to 48 hours (h) after dosing, and the pharmacokinetic (PK) profiles of radioactivity in the blood and plasma were investigated using liquid scintillation counting (LSC)
  - To assess tissue distribution in albino rats (n = 6), animals were euthanized by carbon dioxide inhalation after single oral dosing at 0.5, 2, 4, 8, 24, and 168 h after administration, and whole-body radioluminograms were prepared using a bioimaging analyzer system (BAS)
  - To assess excretion of [^14^C]dersimelagon, urine, feces, and expired air were collected up to 96 h after administration from albino rats (n = 8) housed in metabolic cages. Radioactivity was determined by LSC
  - To assess excretion of radioactivity in bile, the common bile ducts of albino rats were cannulated with polyethylene tubes, and the animals were treated with a single oral dose of [^14^C]dersimelagon. Bile, urine, and feces were collected
    - To study enterohepatic circulation, bile samples of 4 bile duct–cannulated rats were collected up to 8 h after administration and pooled
    - Pooled bile was injected into the duodenum of albino rats, and those animals were bile duct–cannulated in the same manner as the earlier rats. Bile, urine, and feces were collected
- Male Long-Evans pigmented rats (n = 6) aged 7 weeks supplied by the Institute for Animal Reproduction (Kasumigaura, Japan) were used to evaluate tissue distribution following a single oral dose of [^14^C]dersimelagon
  - Following oral dosing of 3 mg/kg [^14^C]dersimelagon, animals were euthanized at 0.5, 2, 4, 8, 24, and 168 h postdose, and the defined tissues were excised
  - Blood was collected from the abdominal aorta using a heparin sodium−treated vacuum tube with injection needle
  - Radioactivity concentrations in the tissues were determined using LSC

*Monkeys*

- Male cynomolgus monkeys aged 3 years (n = 8, supplied by Hamri Co., Ltd. (Koga City, Japan) were used in this study to evaluate the absorption and excretion of [^14^C]dersimelagon following single oral or IV administration
- For oral administration, the dosing formulations were prepared immediately before use at a dose of 3 mg/kg and administered as a 10 mL/kg volume into the stomach via a syringe equipped with an oral catheter
- For IV administration, the dosing formulations were prepared immediately before use at a dose of 1 mg/kg and administered as a 1 mL/kg volume into the medial cutaneous vein using a syringe equipped with an injection needle
- Blood was withdrawn serially from the femoral vein of animals after a single administration of [^14^C]dersimelagon, and the PK profiles of radioactivity concentrations in the blood and plasma were investigated
  - Blood (0.5 mL) was collected at 0.083, 0.25, 0.5, 1, 2, 4, 6, 8, 24, 48, 72, 96, and 120 h after administration using a vacuum blood collection tube treated with heparin sodium
- Urine was collected 0–8, and both urine and feces were collected 8–24, 24–48, 48–72, 72–96, 96–120, 120–144, and 144–168 h after administration, and excretion of radioactivity in the urine or feces was determined
- Radioactivity in each sample was measured using LSC

*Placental Transfer and Milk Secretion in Female Rats*

- Pregnant female Sprague-Dawley (Crl:CD) rats (n = 3) supplied by Charles River Laboratory Japan, Inc. (Yokohama, Japan) were used to assess placental transfer of [^14^C]dersimelagon following single oral administration at day 18 of gestation
  - The dosing formulations were prepared at a dose of 3 mg/kg and administered orally as a 10 mL/kg volume into the stomach via a syringe equipped with an oral tube
  - To perform quantitative whole-body autoradiography, animals were euthanized by carbon dioxide inhalation at 0.5, 2, and 8 h postdose. Radioactivity in 30-μm thick frozen sections of extracted tissues was determined using BAS
  - Just before animals were euthanized, approximately 350 μL of blood was collected from the tail vein using a heparinized capillary tube. Radioactivity (dpm) in each sample was measured using LSC
- Postpartum female Sprague-Dawley (Crl:CD) rats (n = 3) supplied by Charles River Laboratory Japan, Inc. (Yokohama, Japan) were used to assess milk secretion of [^14^C]dersimelagon following single oral administration at postpartum day 12
  - The dosing formulations were prepared at a dose of 3 mg/kg and administered orally as a 10-mL/kg volume into the stomach via a syringe equipped with an oral tube
  - Blood (approximately 250 μL) was collected from the maternal tail vein using a heparinized capillary tube, and milk was collected from the maternal breast or abdominal nipples under anesthesia at each sampling time at 0.5, 2, 4, and 8 h following administration
  - Radioactivity (dpm) in each sample was measured using LSC

**In Vivo Metabolism in Rats and Monkeys**

*Rats*

- The plasma, urine, feces, and bile of male Sprague-Dawley (Crl:CD) rats aged 7 weeks supplied by Charles River Laboratory Japan, Inc. (Yokohama, Japan) were used to perform metabolite analysis following single oral administration of [^14^C]dersimelagon
- Plasma samples from 0.5, 4, and 8 h after administration, and urine, fecal, and bile samples from 0–48 h postdose were collected, and radioactivity of each sample was determined using LSC
- Radiochromatograms were used to identify metabolites derived from dersimelagon, and a percentage of each metabolite (or dersimelagon) was calculated

*Monkeys*

- Plasma, urine, and fecal samples from male cynomolgus monkeys from the absorption and excretion study in monkeys were used to conduct metabolite profiling
- Plasma samples from 2, 4, and 8 h postdose, urine pooled from 0–96 h postdose, and feces pooled from 0–96 h postdose were collected, and radioactivity of each sample was determined using LSC
- Radiochromatograms were used to identify metabolites derived from dersimelagon, and a percentage of each metabolite (or dersimelagon) was calculated

**In Vitro Metabolism in Hepatocytes**

*Mouse*

- [^14^C]dersimelagon free base (5 μmol/L) was incubated for 4 h with male mouse cryopreserved hepatocytes (ICR/CD-1) (pooled from 36 animals) manufactured by BioIVT (Westbury, NY US), and the metabolic profiles were evaluated
- Each sample was analyzed by a high-performance liquid chromatography radioactivity detector (HPLC-RAD) system, and radiochromatograms were obtained

*Rat, Monkey, and Human*

- [^14^C]dersimelagon free base (5 μmol/L) was incubated for 4 h with cryopreserved hepatocytes from male Sprague-Dawley rats (pooled from 30 animals), male cynomolgus monkeys (pooled from three animals), and male and female humans (pooled from 25 males and 25 females) manufactured by BioIVT (Westbury, NY US), and the metabolic profiles were evaluated
- Each sample was analyzed by an HPLC-RAD system, and radiochromatograms were obtained

**Metabolism in Healthy Adults**

- Plasma and fecal homogenates from six healthy participants in the phase 1 study were pooled to prepare plasma samples and fecal homogenate, respectively. The plasma and fecal samples were extracted with 0.1% formic acid in ethanol
- Chromatographic separation was achieved with an HPLC column (InertSustain C18, 3 μm, 4.6 mm I.D. × 250 mm L, GL Sciences [Torrance, CA, US]) with a gradient program, which combined two solvents (A and B) at 55 °C. Solvent A consisted of 10 mmol/L ammonium acetate/formic acid (100:0.5, v/v) and Solvent B consisted of acetonitrile/methanol/10 mmol/L ammonium acetate/formic acid (70:20:10:0.5, v/v/v/v)
- The HPLC flowrate was 1.0 mL/min. The mobile phase composition started at 30% B and was maintained for 10 min, followed by an increase to 45% B from 10 to 65 min, 55% B from 65 to 65.1 min, and 100% B 65.1 to 85 min, followed by a decrease to 30% B from 85 to 100 min
- The HPLC eluate was fractionated with a fraction collector, and the radioactivity was measured using an accelerator mass spectrometer (plasma sample) or liquid scintillation counter (fecal sample)
- Based on radiochromatogram of the pooled plasma samples (0–48 h) and fecal samples (0–168 h), percentages of dersimelagon and its metabolites to the total radioactivity in the sample calculated from the radiochromatogram were converted to area under the radioactivity concentration in plasma–time curves or excretion of radioactivity in feces
- Percentages of radioactive peaks were calculated using the following equations:
  - Percentage of radioactivity in sample = radioactivity corresponding to each peak/total of radioactivity corresponding to detected peaks × recovery through sample processing × 100
  - Fecal excretion of dersimelagon or its metabolite (% of dose) = percentage of radioactivity in sample / 100 × excretion of total radioactivity

**Supplemental Table 1.** In vivo pharmacokinetic parameters of radioactivity in plasma after administration of [^14^C]dersimelagon to rats and monkeys

| Species,  route (dose) | C_max_  (ng eq./mL) | T_max_  (h) | AUC_0–∞_  (ng eq.·h/mL) | t_1/2_ (h) | CL (mL/h/kg) | V_ss_  (mL/kg) |
| --- | --- | --- | --- | --- | --- | --- |
| Rats |  |  |  |  |  |  |
| IV (2 mg/kg) | – | – | 904 | 2.38 | 2259.8 | 3388 |
| PO (3 mg/kg) | 151.98 | 0.50 | 478 | 3.86 | – | – |
| Monkeys |  |  |  |  |  |  |
| IV (1 mg/kg) | – | – | 2295.5 | 11.09 | 459.9 | 2010.5 |
| PO (3 mg/kg) | 315.63 | 1.50 | 1915.3 | 21.40 | – | – |

Data are expressed as means of four animals.

AUC_0−∞_, area under the concentration-time curve from time 0 extrapolated to infinity; CL, clearance; C_max_, maximum observed concentration; IV, intravenously; PO, orally; t_1/2_, apparent terminal elimination half-life; T_max_, time to C_max_; V_ss_, steady-state volume of distribution.

**Supplemental Table 2.** Tissue distribution of radioactivity concentration (ng eq./g tissues as dersimelagon free base) after administration of [^14^C]dersimelagon to rats

| Species, tissue | 30 min | 2 h | 4h | 8 h | 24 h | 168 h |
| --- | --- | --- | --- | --- | --- | --- |
| Albino rats |  |  |  |  |  |  |
| Adrenal gland | 569.40 | 98.87 | 49.84 | NS | NS | NS |
| Blood | 67.11 | 23.54 | NS | NS | NS | NS |
| Bone | 19.76 | 11.16 | NS | NS | NS | NS |
| Bone marrow | 104.71 | 41.59 | NS | NS | NS | NS |
| Brown fat | 90.49 | 44.26 | BLQ | NS | NS | NS |
| Cerebrum | BLQ | BLQ | NS | NS | NS | NS |
| Epididymis | 16.79 | 31.45 | NS | NS | NS | NS |
| Eyeball | BLQ | BLQ | NS | NS | NS | NS |
| Fat | 28.47 | 14.12 | NS | NS | NS | NS |
| Gastric contents | AUQ | AUQ | AUQ | BLQ | NS | 31.88 |
| Harderian gland | 80.33 | 168.38 | 52.77 | 37.43 | NS | NS |
| Heart | 176.82 | 64.92 | NS | NS | NS | NS |
| Kidney | 361.97 | 119.59 | 35.10 | 8.44 | NS | NS |
| Large intestinal contents | NS | NS | NS | AUQ | 9150.08 | 64.46 |
| Large intestine | 108.80 | 56.50 | 9.77 | 4890.39 | 16.94 | NS |
| Liver | 12169.84 | 3462.50 | 1612.37 | 745.62 | BLQ | NS |
| Lung | 111.85 | 49.91 | 8.79 | NS | NS | NS |
| Mandibular gland | 220.64 | 77.22 | 17.59 | NS | NS | NS |
| Medulla oblongata | BLQ | BLQ | NS | NS | NS | NS |
| Mesenteric lymph nodes | 93.84 | 384.20 | 297.07 | 35.46 | NS | NS |
| Pancreas | 270.66 | NS | NS | NS | NS | NS |
| Pituitary gland | 176.92 | 91.34 | 38.11 | NS | NS | NS |
| Prostate gland | 38.64 | 121.47 | 13.68 | NS | NS | NS |
| Skeletal muscle | 74.22 | 38.61 | NS | NS | NS | NS |
| Skin | 31.52 | 8.47 | NS | NS | NS | NS |
| Small intestinal contents | AUQ | AUQ | AUQ | 11301.58 | 167.42 | 15.00 |
| Small intestine | 1685.82 | 2235.51 | 8223.06 | 397.93 | 38.86 | NS |
| Spleen | 189.66 | 91.29 | 30.36 | NS | NS | NS |
| Stomach | 6153.11 | 3213.50 | 141.35 | 15.76 | NS | NS |
| Testis | BLQ | BLQ | NS | NS | NS | NS |
| Thymus | 33.55 | 48.97 | NS | NS | NS | NS |
| Thyroid gland | 166.75 | 66.86 | NS | NS | NS | NS |
| Urinary bladder | 39.65 | 103.58 | NS | NS | NS | NS |
| Urine in bladder | 16.27 | 147.84 | NS | NS | NS | NS |
| Plasma (by LSC) | 83.78 | 32.52 | 9.51 | 3.09 | ND | ND |
| Blood (by LSC) | 52.42 | 19.96 | 6.48 | 2.40 | ND | ND |
| Pigmented rats |  |  |  |  |  |  |
| Plasma | 189.36 | 32.77 | 25.58 | 5.50 | 2.47 | ND |
| Blood | 122.32 | 22.10 | 17.97 | 2.66 | 2.16 | ND |
| Eyeball | 14.34 | 5.79 | 8.06 | 4.15 | 2.79 | 1.41 |
| Liver | 19744.35 | 20562.02 | 613.26 | 762.72 | 11.09 | 1.32 |
| Kidney | 411.62 | 81.17 | 63.18 | 7.97 | 1.50 | 0.67 |
| Skin (pigmented) | 45.03 | 13.30 | 13.94 | 2.67 | 1.36 | ND |
| Skin (white) | 43.44 | 16.32 | 16.25 | 2.61 | 2.26 | ND |

Data are expressed as the value of one animal.

AUQ, above upper limit of quantification (16291.04); BLQ, below lower limit of quantification (8.28); h, hours; LSC, liquid scintillation counting; min, minutes; ND, not detected; NS, not specified.

**Supplemental Table 3.** Tissue distribution of radioactivity concentration (ng eq. of dersimelagon/g tissue) after administration of [^14^C]dersimelagon to pregnant rats on day 18 of pregnancy

| Tissue | 30 min | 2 h | 8 h |
| --- | --- | --- | --- |
| Amniotic fluid | BLQ | BLQ | NS |
| Blood | 270.85 | 70.94 | BLQ |
| Brain | BLQ | BLQ | NS |
| Fetal blood | NS | NS | NS |
| Fetal brain | NS | NS | NS |
| Fetal heart | NS | NS | NS |
| Fetal kidney | NS | NS | NS |
| Fetal liver | NS | NS | NS |
| Fetal lung | NS | NS | NS |
| Fetus (whole body) | 9.47 | BLQ | BLQ |
| Heart | 632.71 | 216.09 | 12.14 |
| Liver | AUQ | 5425.09 | 1416.67 |
| Lung | 494.03 | 141.79 | 25.36 |
| Mammary gland | 207.31 | 64.39 | 11.13 |
| Ovary | 250.27 | 111.40 | 24.19 |
| Placenta | 286.02 | 61.78 | 12.68 |
| Renal cortex | 762.13 | 231.31 | 29.23 |
| Renal medulla | 363.13 | 184.84 | 12.09 |
| Uterus | 141.01 | 65.48 | 9.11 |
| Plasma (by LSC) | 231.06 | 95.47 | 10.75 |
| Blood (by LSC) | 151.18 | 57.77 | 6.66 |

Data are expressed as the value of one animal.

AUQ, above upper limit of quantification (16469.16); BLQ, below lower limit of quantification (8.37); h, hours; LSC, liquid scintillation counting; min, minutes; NS, not specified.

**Supplemental Table 4.** Cumulative excretion of radioactivity after administration of [^14^C]dersimelagon to rats, monkeys, and bile duct–cannulated rats

| Species, |  | Cumulative excretion of radioactivity (% of dose) | | | | | |
| --- | --- | --- | --- | --- | --- | --- | --- |
| route (dose) | Time (h) | Bile | Urine | Feces | Expired air | GI contents | Carcass |
| Intact rats |  |  |  |  |  |  |  |
| IV (2 mg/kg) | 0–96 | – | 0.9 | 96.4 | 0.0 | – | 0.0 |
| PO (3 mg/kg) | 0–96 | – | 0.7 | 98.2 | 0.0 | – | 0.1 |
| Intact monkeys |  |  |  |  |  |  |  |
| IV (1 mg/kg) | 0–168 | – | 0.7 | 99.2 | – | – | – |
| PO (3 mg/kg) | 0–168 | – | 0.2 | 100.6 | – | – | – |
| Bile duct–cannulated rats |  |  |  |  |  |  |  |
| PO (3 mg/kg) | 0–48 | 63.6 | 0.2 | 33.3 | – | 0.8 | 0.2 |

Data are expressed as the mean of four animals. The fraction absorbed from the digestive tract was calculated as the sum of cumulative excretion of radioactivity in bile and urine and the residual radioactivity in the carcass after an oral dose to bile duct–cannulated rats.

–, not determined; GI, gastrointestinal; h, hours; IV, intravenously; PO, orally.

**Supplemental Table 5.** In vivo metabolism of [^14^C]dersimelagon in rats and monkeys (% total radioactivity)

| Species, metabolite | Plasma | Urine | Feces | Bile |
| --- | --- | --- | --- | --- |
| Rats |  |  |  |  |
| Time point | 4 h | 0–48 h | 0–48 h | 0–48 h |
| M02^†^ | – | – | – | 2.0 (1.3) |
| M06a^‡^ | 5.9 (0.62) | 9.6 (0) | 1.6 (1.5)^§^ | 77.3 (51.0) |
| M07 | – | 3.8 (0) | 7.4 (6.8) | 2.1 (1.4) |
| M08a^‡^ | – | – | 2.8 (2.6) | 1.4 (0.9) |
| Dersimelagon | 68.0 (7.17) | 26.7 (0.1) | 70.9 (65.5) | 7.9 (5.2) |
| Monkeys |  |  |  |  |
| Time point | 2 h | 0–96 h | 0–96 h |  |
| M02b^¶^ | – | 3.3 (0) | 4.6 (4.5) | – |
| M06a^‡^ | 9.1 (21.91) | 15.5 (0) | – | – |
| M07 | – | 6.4 (0) | 10.0 (9.8) | – |
| M08a^‡^ | – | 15.6 (0) | 12.1 (11.9) | – |
| Dersimelagon | 90.0 (216.73) | 18.4 (0) | 51.3 (50.4) | – |

Plasma, urine, feces, and bile were collected after a single oral administration of [^14^C]dersimelagon to male rats or male cynomolgus monkeys (3 mg/kg). Values in parentheses are concentration (ng eq. of dersimelagon/mL, plasma) or % of dose (urine, feces, and bile).

^†^Mixture of M02a, M02b, M02c-1, M02c-2, and M02d.

^‡^Including minor components M06b (with M06a) and M08b (with M08a).

^§^M06c as a main component.

*^¶^*Including minor components (M02a, M02c-1, and M02c-2 in urine, and M02c-1 and M02c-2 in feces).

–, not determined; h, hours;

**Supplemental Table 6.** In vitro metabolism of [^14^C]dersimelagon free base in mouse, rat, monkey, and human hepatocytes
(% total radioactivity)

| Metabolite | Mouse hepatocytes | Rat hepatocytes | Monkey hepatocytes | Human hepatocytes |
| --- | --- | --- | --- | --- |
| M02^†^ | – | 6.4 | 2.4 | 1.7 |
| M06a^‡^ | 2.4 | 38.5 | 38.5 | 43.0 |
| M07 | – | 2.7 | – | – |
| M08a^‡^ | 1.1 | 1.5 | 3.2 | 2.6 |
| Dersimelagon | 92.4 | 34.6 | 43.9 | 45.8 |

5 μmol/L of [^14^C]dersimelagon free base was incubated for 4 hours.

^†^M02 was a mixture of M02a and M02b.

^‡^Including minor components M06b (with M06a) and M08b (with M08a).

–, not determined.
